# A Solvothermal Synthesis of TiO_2_ Nanoparticles in a Non-Polar Medium to Prepare Highly Stable Nanofluids with Improved Thermal Properties

**DOI:** 10.3390/nano8100816

**Published:** 2018-10-10

**Authors:** Teresa Aguilar, Ivan Carrillo-Berdugo, Roberto Gómez-Villarejo, Juan Jesús Gallardo, Paloma Martínez-Merino, José Carlos Piñero, Rodrigo Alcántara, Concha Fernández-Lorenzo, Javier Navas

**Affiliations:** 1 Departamento de Química Física, Facultad de Ciencias, Universidad de Cádiz, E-11510 Puerto Real (Cádiz), Spain; ivan.carrillo@uca.es (I.C.-B.); roberto.gomezvi@uca.es (R.G.-V.); jj.gallardo@uca.es (J.J.G.); paloma.martinez@uca.es (P.M.-M.); rodrigo.alcantara@uca.es (R.A.); concha.fernandez@uca.es (C.F.-L.); 2 Departamento de Ciencias de los Materiales, Ingeniería Metalúrgica y Química Inorgánica, Facultad de Ciencias, Universidad de Cádiz, E-11510 Puerto Real (Cádiz), Spain; josecarlos.pinero@uca.es

**Keywords:** nanofluids, nanoparticles, thermal properties, heat transfer process, concentrating solar power

## Abstract

Nanofluids are systems with several interesting heat transfer applications, but it can be a challenge to obtain highly stable suspensions. One way to overcome this challenge is to create the appropriate conditions to disperse the nanomaterial in the fluid. However, when the heat transfer fluid used is a non-polar organic oil, there are complications due to the low polarity of this solvent. Therefore, this study introduces a method to synthesize TiO_2_ nanoparticles inside a non-polar fluid typically used in heat transfer applications. Nanoparticles produced were characterized for their structural and chemical properties using techniques such as X-ray Diffraction (XRD), Raman spectroscopy, Transmission Electron Microscopy (TEM), Fourier Transform Infrared (FTIR) spectroscopy, and X-ray photoelectron spectroscopy (XPS). The nanofluid showed a high stability, which was analyzed by means of UV-vis spectroscopy and by measuring its particle size and ζ potential. So, this nanofluid will have many possible applications. In this work, the use as heat transfer fluid was tested. In this sense, nanofluid also presented enhanced isobaric specific heat and thermal conductivity values with regard to the base fluid, which led to the heat transfer coefficient increasing by 14.4%. Thus, the nanofluid prepared could be a promising alternative to typical HTFs thanks to its improved thermal properties and high stability resulting from the synthesis procedure.

## 1. Introduction

Nanofluids are a colloidal suspension on nanosized materials in a base fluid which was for the first time reported by Choi et al. in 1995 [1]. Generally, the addition of nanoparticles yields an enhancement of thermal conductivity that depends on the volume fraction of nanomaterials, particle size, nature of the material, etc. So, nanofluids have been shown to be an interesting alternative to the typical heat transfer fluids in several applications, such as the cooling of electronic devices [2,3] and solar energy [4].

One of the most important challenges in colloidal suspension technology is the length of time that the colloid remains stable. In the case of nanofluids, stability is an important factor to take into account because their thermal properties depend on the amount of nanoparticles in the base fluid that are able to transport heat [5,6,7]. In general, all systems are characterized to reach their minimum energy state, so the normal tendency of nanoparticles in a nanofluid is to agglomerate due to van der Waals forces of attraction. Several physical treatment techniques have been used to disperse nanoparticles well in a fluid, including probe ultrasonication, bath ultrasonication, magnetic stirring, and high pressure homogenization [5,8,9]. Different strategies have been used that involve chemical changes on the surface of the nanoparticles, such as steric or electrostatic stabilization by varying the pH of the system or by the addition of different surfactants [8,10,11,12]. To date, two different methods have been reported for preparing nanofluids: the single-step and the two-step method. The single-step method involves the in-situ synthesis of the nanoparticles in the base fluid. On the other hand, in the two-step method, the nanoparticles are first synthesized, controlling their shape, size, etc., and then dispersed into the base fluid to obtain a colloidal suspension, typically by means of sonication. However, several authors have reported that this method usually produces unstable colloidal suspensions in which the nanoparticles agglomerate and settle after a few days [13,14]. Several authors have used a surfactant to prevent the agglomeration and later precipitation of the nanoparticles, but surfactants can affect the thermal properties of the fluid. For example, Xuan et al. [15] studied the effect of the loading of SBDS in Cu-nanofluids prepared with water as the base fluid, reporting a decrease in thermal conductivity among the nanofluids with a higher concentration of surfactant.

To reduce the agglomeration and consequent sedimentation of nanoparticles in a base fluid, the one-step method mentioned above may be a good alternative. In the one-step method, nanoparticles are simultaneously synthesized and dispersed in the liquid. This method is able to improve the stability of nanofluids while cutting production costs. Examples of the one-step method include inert gas condensation [16], chemical reduction [17], pulsed wire evaporation [18], and the arc-submerged nanoparticles synthesis system (ASNSS) [19]. However, the one-step method is typically used to synthesize metal or metal oxides in water [17,18,19,20] or ethylene glycol [16]. The synthesis of nanomaterials in low polar fluids such as synthetic oil is not common due to the complexity of the process.

Therefore, this study presents a new, one-step method for synthesizing TiO_2_ nanoparticles in a non-polar thermal oil typically used in concentrating solar power (CSP) plants. TiO_2_-based nanofluids have been reported as an interesting heat transfer fluid in several applications [21,22]. The novelty of this synthesis method is that the TiO_2_ is prepared using a procedure that is very similar to a hydrolysis reaction in the absence of water, with the synthesis taking place in a non-polar medium. To our knowledge, this is the first time that this methodology has been applied to prepare nanofluids.

## 2. Materials and Methods 

**Preparation of the nanofluids.** In this study, the synthesis of TiO_2_ has been performed by a solvothermal reaction in the absence of water, using a thermal oil as the medium and benzylic alcohol as the reagent, which has already been proven to be a versatile reagent for the synthesis of transition metal oxide nanoparticles with good control over the particle size, shape, and crystallinity [23]. Furthermore, this synthesis cannot be performed using water as the hydrolyzing agent because water and the thermal oil used are not miscible with each other. Thus, a thermal oil consisting of the eutectic mixture of biphenyl (26.5%) and diphenyl oxide (73.5%) (Dowthern A, supplied by The Dow Company^©^, Midland, MI, USA) was added to a Teflon cup, which was then placed in a glove box at room temperature to rule out the possible influence of high humidity in the environment. This eutectic mixture is the base fluid of the nanofluid prepared. A volume of 3.5 mL of benzylic alcohol (% purity, Panreac^©^, Barcelona, Spain) was added and mixed with 96 mL of thermal oil. Benzylic alcohol was chosen due its good versatility, solubility in ether (the main solvent of HTF used in this study), and the similar density of both liquids, which could prevent the two phases from separating. Next, 0.5 mL of titanium isopropoxide (TTIP, Ti(iOPr)_4_, Panreac^©^, Barcelona, Spain) was added drop by drop to the solution. The reaction was carried out into an autoclave with constant magnetic stirring at 473 K for 48 h with inert atmosphere (N_2_). The colloidal suspension obtained from the solvothermal synthesis was the nanofluid.

The reaction can be defined as a non-aqueous sol-gel process. In a typical aqueous sol-gel reaction, the oxygen for the formation of oxide compounds is supplied by water molecules. In this study, in the absence of water, the oxygen for nanoparticle formation is provided by the benzylic alcohol. The reaction proposed would be:

$$\;Ti{\left(iOPr\right)}_{4}+\;xC_{7}H_{7}OH\to Ti{\left(iOPr\right)}_{4-x}{\left(C_{7}H_{7}O\right)}_{x}+xC_{3}H_{7}OH\;$$

 ≡Ti−O−R+R−O−Ti≡ → ≡Ti−O−Ti≡ + R−O−R 

The reaction can occur in two steps. First, benzylic alcohol reacts with TTIP to generate an intermediate product, where isopropyl chains are substituted by phenyl groups. Next, the intermediate product condenses to form a Ti–O–Ti bond, in order to obtain a TiO_2_ structure, and different ether molecules are formed, as has been reported previously [24] and analytically verified below.

**Characterization of nanoparticles.** To characterize the synthesized TiO_2_ nanoparticles, several instrumental techniques were used to determine their structure and crystalline phase. Previously, the nanoparticles were extracted from the nanofluid synthesized. To extract the nanoparticles, the nanofluid was centrifuged and the supernatant discarded, resulting in a sludge, which was dried in an oven at 353 K for 48 h. The synthesized nanoparticles were compared with commercial TiO_2_ (Degussa P25, supplied by INSCX^©^, Cheshire, England). A structural study was carried out using an X-ray powder diffractometer (Bruker^©^, Billerica, MA, USA, model D8Advanced A25 Davinci) with Cu-Kα radiation. The patterns were recorded in the range between 10 and 75° in 2θ and the scan conditions were a resolution of 0.02°, 40 kV, and 40 mA. The crystal structure was further analyzed by Raman spectroscopy using iRaman, supplied by MicroBeam^©^ (Madrid, Spain), equipped with a laser diode supplied by B&W TecInk^©^ (Herisau, Switzerland) with emission at 785 nm. In addition, transmission electron microscopy (TEM) measurements of the TiO_2_ nanoparticles were performed using the beam of a JEOL (Akishima, Tokyo, Japan) 2100 instrument, with an acceleration voltage of 200 kV. For the TEM characterization, the samples were prepared in a way that one drop of the dispersion of as-synthesized nanoparticles in the base fluid was deposited onto a copper grid covered by an amorphous carbon film. In addition, FT-IR spectra were measured using a Bruker^©^ (Billerica, MA, USA) spectrometer, model Tensor37. The data were recorded from 400 to 4000 cm^−1^ and at a resolution of 2 cm^−1^. Finally, X-ray photoelectron spectroscopy (XPS) was used to analyze the oxidation state and the chemical state bonding of the elements in the samples. The XPS spectra were recorded using a Kratos (Kyoto, Japan) Axis UltraDLD spectrometer, with monochromatized Al Kα radiation (1486.6 eV), 20 eV pass energy, and an accuracy of 0.1 eV. Electrostatic charging effects were stabilized with the help of a specific device developed by Kratos (Kyoto, Japan).

**Characterization of nanofluids.** The nanofluids were analyzed to determine the stability of the nanoparticle suspension and whether their thermal properties were enhanced due to the formation of TiO_2_ nanoparticles in the base fluid. Several techniques were used to assess the stability of the nanofluids. UV-vis spectra were recorded and particle size and ζ potential measurements performed. The UV-vis spectra were studied using equipment assembled in our laboratory. The system consists of a halogen lamp, model DH-2000-BAL, supplied by Ocean Optics^©^ (Amersham, United Kingdom), as the illumination source, and a USB2000+ spectrometer supplied by OceanOptics^©^ (Amersham, United Kingdom). The dynamic light scattering (DLS) technique was used to measure the size of the nanoparticles. Furthermore, ζ potential provides information about the charge surrounding the nanoparticles by measuring their electrophoretic mobility in a base fluid through the application of an electric field. Both measurements were performed using a Zetasizer Nano ZS supplied by Malvern Instruments Ltd^©^ (Amersham, United Kingdom). Due to the low dielectric constant of the base fluid, the nanoparticles presented low mobility, so the Huckel model was selected to measure ζ potential, and a voltage of 120 V was applied to boost the movement of the nanoparticles in the base fluid.

To characterize the efficiency of the TiO_2_-nanofluid inside a tube in heat transfer applications, measurements were taken of its density, dynamic viscosity, isobaric specific heat, and thermal conductivity. The density was estimated using a pycnometer and a thermal bath supplied by Selecta^©^ (Barcelona, Spain), which controlled the temperature of the nanofluid. In addition, a Malvern^©^ (Amersham, United Kingdom). SV-10 viscometer was used to measure dynamic viscosity. This system uses vibrating paddles to measure the sample viscosity, which vibrate at a frequency a displacement characteristic. The system registers the reduction in displacement due to the sample in which the paddles are immersed. The measurements were performed in a thermostatically controlled cell holder recirculating water. All measurements were performed in triplicate. Moreover, isobaric specific heat was measured using a temperature modulated differential scanning calorimeter (TMDSC), supplied by TA Instruments^©^ (Milford, MA, USA), model Q-20. The program generated to perform the measurements can be summarized as: (a) an initial isothermal step for 10 min at 341 K to eliminate contaminants; (b) the sample was equilibrated at 301 K and then the temperature was ramped up to 391 K at a rate of 1 K/min; (c) a modulation function with an amplitude of ±1 K and a period of 120 s was used to study the range of temperatures of interest; and (d) finally, the sample was cooled at 1 K/min. Finally, thermal conductivity was measured by using the light flash analysis technique (LFA 1600 equipment, supplied by Linseis Thermal Analysis^©^, Selb, Germany). This technique measures thermal diffusivity, which is the thermo-physical property that defines the heat propagation rate by conduction during changes of temperature. So, thermal conductivity can be calculated according to

*k*(T) = *ρ*(T)·*C_P_*(T)·*α*(T), (1)

where k is thermal conductivity, ρ is the density, $C_{p}$ is the isobaric specific heat, and α is thermal diffusivity.

## 3. Results and Discussions

### 3.1. Material Characterization

**Figure 1:**
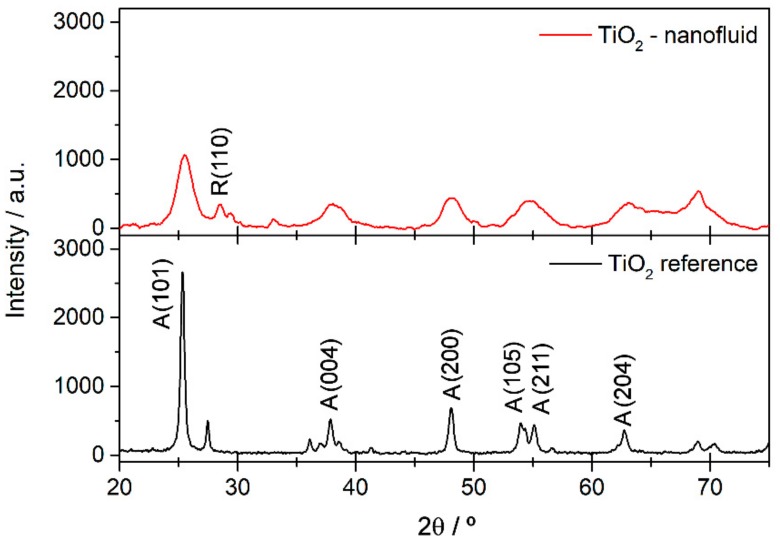
X-ray Diffraction (XRD) patterns for TiO_2_ synthesized and commercial TiO_2_ nanoparticles used as a reference.

The crystalline phases and crystallinity of the synthesized nanoparticles were analyzed by X-ray diffraction. The patterns of commercial TiO_2_ and the synthesized sample are shown in Figure 1. The synthesized TiO_2_ nanoparticles show low crystallinity, observed from the low intensity of the diffractogram compared with the reference of TiO_2_ anatase phase in the pattern below, and also from the presence of broad peaks in the diffractogram obtained. The main reflections of the planes of anatase phase (A) according to the reference TiO_2_ (JCPDS 21-1272) can be identified, and they are highlighted in Figure 1. It is also possible to observe the main peak of rutile phase (R), which is assigned to the reflection of the (110) plane, but it is shifted from the typical position at about 27.4°. This may be due to micro-strain of the rutile lattice, as is shown and confirmed by the TEM results below. In addition, the low crystallinity may be a result of physical and chemical defects in the structure, such as oxygen vacancies, which can be related to species absorbed onto the surface of the TiO_2_ due to the points with high reactivity. 

**Figure 2:**
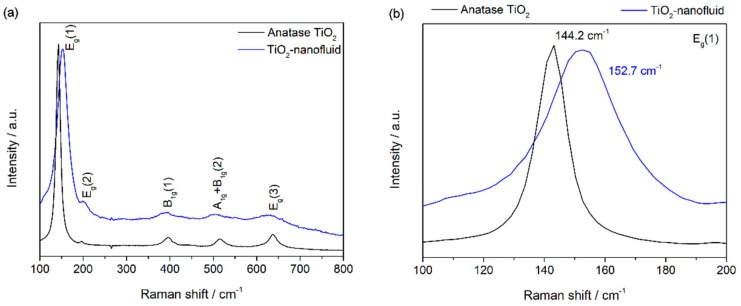
Raman spectroscopy of TiO_2_ synthesized (**a**) and the reference of TiO_2_ anatase structure (**b**).

Raman spectroscopy was used to identify different structures. Figure 2a shows the Raman spectra collected from the nanoparticles of the synthesized sample and a reference of TiO_2_ anatase phase in the region of 100–800 cm^−1^. Anatase TiO_2_ shows a tetragonal structure with six Raman active modes (A_1g_ + 2B_1g_ + 3E_g_). The Raman spectrum of an anatase single crystal has been widely studied [25,26], and the six allowed modes are known to appear at 144 cm^−1^ (E_g_), 197 cm^−1^ (E_g_), 399 cm^−1^ (B_1g_), 513 cm^−1^ (A_1g_), 519 cm^−1^ (B_1g_), and 639 cm^−1^ (E_g_). All the active modes can be identified in the spectrum registered for the synthesized TiO_2_ nanoparticles (see Figure 2a). Focusing on the main vibration mode for anatase phase, E_g_, shown in Figure 2b, a shift towards higher wavenumbers is observed. This displacement may occur because of low crystallinity and the presence of oxygen vacancies in the TiO_2_ structure, as has been reported previously [27].

**Figure 3:**
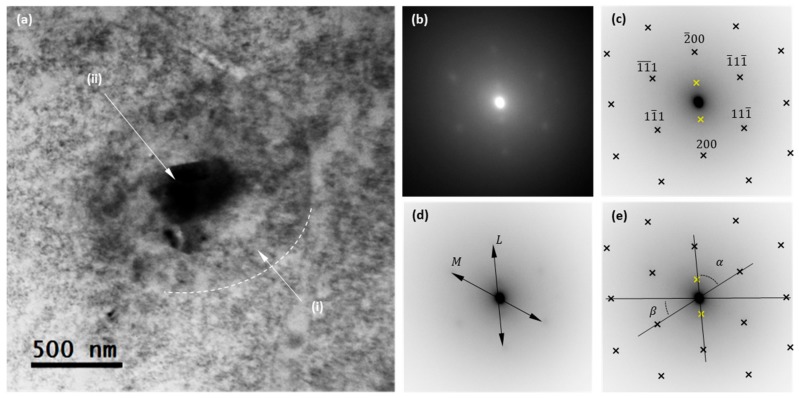
(**a**) Transmission Electron Microscopy (TEM) bright field overview of a single nanoparticle; dark contrast indicates the TiO_2_ core, while the weaker dark contrast surrounding the nuclei reveals an outer carbon-made shell. (**b**) Selected area electron diffraction patterns (SAED) obtained in the nanoparticle. Superlattice spots are evidenced. (**c**) Indexed diffraction pattern, contrast inverted. (**d**) Definition of the L and M axis, used to calculate the zone axis. (**e**) Definition of the angles α and 𝛽, used to calculate the zone axis.

The structures in the sample were determined using transmission electron microscopy (TEM). Figure 3a shows a TEM bright field micrograph of a representative TiO_2_ nanoparticle, where a core-shell structure is evidenced. White arrows are used in Figure 3a to highlight core and shell regions, where label (i) indicates the carbon-made “shell” region. As the shell region appears as a weak dark contrast in the TEM image, a dashed white line has been superimposed to guide the eyes around the edge of the nanoparticle. This weak contrast observed between this shell region and the amorphous film that supports the nanoparticle can be explained by the fact that both are carbon-made. In turn, label (ii) indicates the core region of the TiO_2_ nanoparticle, which appears as a dark contrast in the TEM image.

The TEM images show that the nanoparticle has a uniform shape, presenting a spherical symmetry with an outer diameter between 1.3 and 1.1 μm (measurements taking into account the outer carbon shell), while the inner diameter (that of the TiO_2_ core) is shown to be less uniform, ranging from 300 to 450 nm. But the size of this nanoparticle is not representative of the system. The analysis of the distribution size was performed using the DLS technique and is shown below.

Selected area electron diffraction patterns (SAED) were recorded at the TiO_2_ core (region (ii)). The resulting image is shown in Figure 3b, with there being evidence of various diffraction spots. Indeed, besides the fundamental fcc reflections, the spots close to the transmitted beam circle in the SAED pattern are evidence of a superlattice structure. According to this, the isolated nanoparticle corresponds to a crystalline structure consisting of two phases. Moreover, this behavior demonstrates the crystallinity of the nanoparticle’s core.

To clarify the picture, Figure 3c shows an inverted contrast image of the recorded SAED in which dark crosses are used to highlight the positions of the main reflections of the first lattice, while yellow crosses are used to highlight the two reflections of the second lattice. To determine the zone axis of the first lattice, the *L* and *M* axes are defined in the SAED, as presented in Figure 3d. The calculated *L*/*M* ratio is: $L/M=1.09\approx 2/\sqrt{3}$, which is consistent with an fcc (011) zone axis. In turn, the experimental values of angles α and 𝛽, as defined in Figure 3e, are found to be 56.67° and 32.45° respectively, very close to the theoretical values of 54.74° and 35.26°. The discrepancies between the experimentally measured values and the theoretical ones can be due to a slight micro-strain of the rutile lattice. The calculated values for the lattice constants are a = b = 3.7Å and c = 2.9 Å.

A second lattice is evidenced in the SAED and highlighted in Figure 1c with yellow crosses. These two reflections are hardly visible and corroborate the two-phase character of the TiO_2_ sample, with anatase phase being the most probable candidate, as can be deduced from the ratio of the reflections of both lattices. These results are in good agreement with those obtained from XRD.

**Figure 4:**
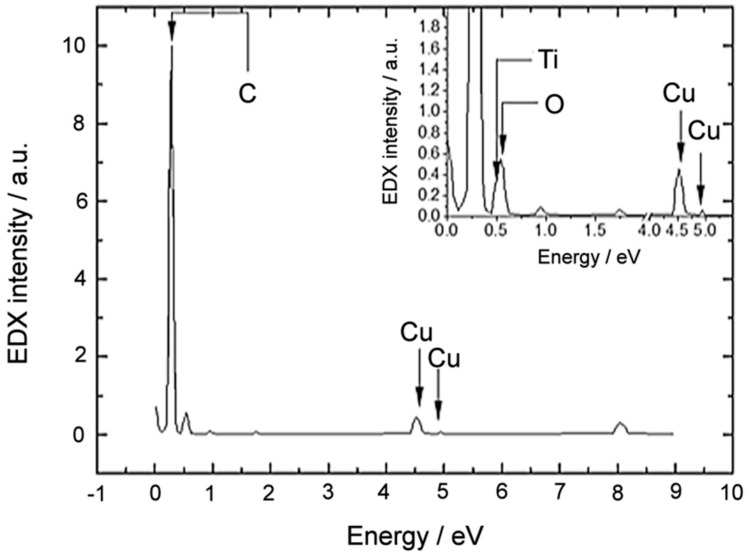
Energy-dispersive X-ray (EDX) spectra acquired right in the core of the nanoparticle.

Finally, to corroborate the composition of the nanoparticle, EDX (Energy-dispersive X-ray) spectra were acquired right in the core region. The corresponding spectrum is presented in Figure 4; it presents Ti- and O-related peaks that are labelled and highlighted with dark arrows. Furthermore, a C-related peak reveals the nature of the outer shell of the nanoparticle. The presence of Cu-related peaks is attributed to the Cu-grid of the TEM specimen holder.

**Figure 5:**
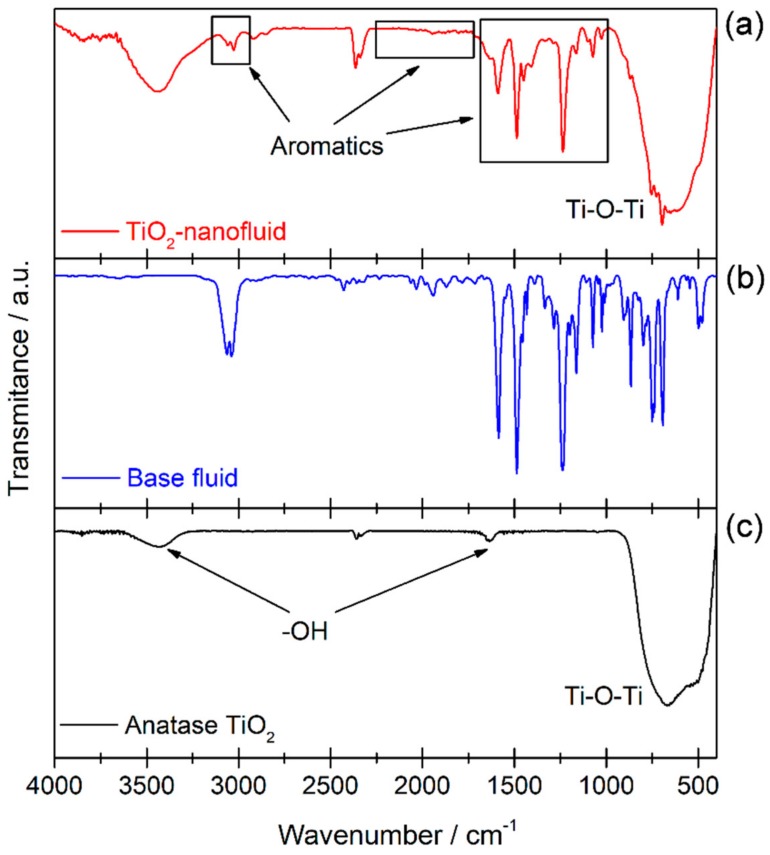
Fourier Transform Infrared (FTIR) spectra of TiO_2_ sample extracted from the nanofluid (**a**), base fluid (**b**), and reference TiO_2_ anatase (**c**).

As detailed above, the main aim of this study is the synthesis of TiO_2_ nanoparticles in a non-polar medium to obtain a nanofluid with good dispersion and a good stability, probably thanks to the presence of phenyl groups surrounding the nanoparticles. Thus, FTIR spectroscopy was used to confirm the presence of TiO_2_ and other organic species. Figure 5 shows FTIR transmission spectra of the TiO_2_ sample (a), the base fluid used to prepare the nanofluid (b), and a reference of TiO_2_ with anatase phase (c) to compare with the nanoparticles formed. A broad band is observed between 1000–400 cm^−1^, which is the typical zone for the stretching vibration of Ti–O bonds and bending vibration of Ti–O–Ti [28,29]. Also, a broad band can be seen around 3400 cm^−1^, assigned to OH bonds [29,30]. Furthermore, the FTIR spectrum for the synthesized TiO_2_ shows the typical peaks for aromatic species (in squares in the spectrum shown in Figure 5a). Comparing it with the spectrum obtained for the base fluid (see Figure 5b), the presence of base fluid molecules is observed, probably around the surface of TiO_2_ nanoparticles. The adsorption of species onto the surface of the TiO_2_ could be due to oxygen vacancies, which are known to lead to distortion of the electron clouds in order to balance the charge deficit generated by the vacancy, which generates adsorption centers. In these centers, molecules of the base fluid or benzylic alcohol are probably joined to TiO_2_ nanoparticles, generating the signals for aromatic bonds on the FTIR spectrum. 

**Figure 6:**
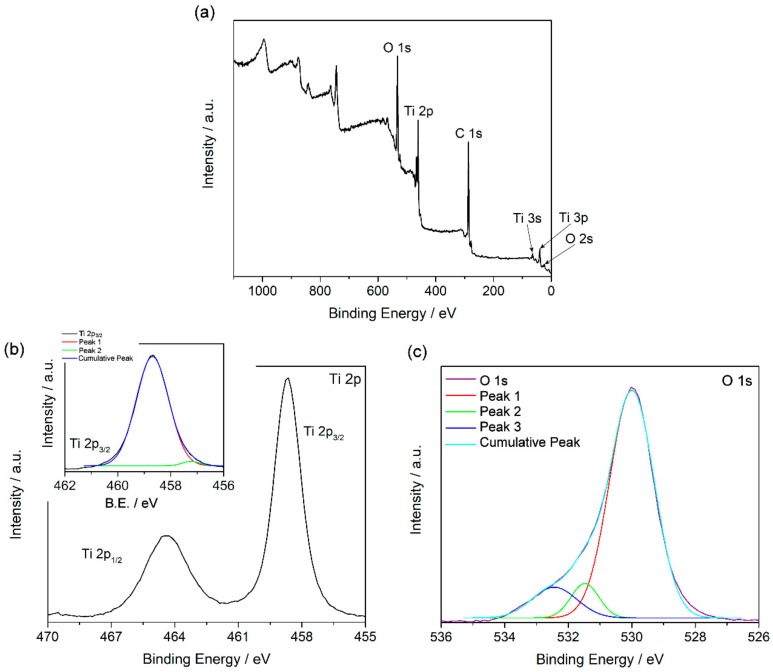
General X-ray photoelectron spectroscopy (XPS) spectrum for TiO_2_ nanoparticles synthesized (**a**), signals for Ti 2p (**b**), and O 1s (**c**) obtained from XPS measurements.

XPS measurements were performed to analyze the oxidation state and the chemical state bonding of the elements composing the sample. Figure 6a shows the general spectrum obtained and the assignation of the peaks found. In detail, Figure 6b shows the Ti 2p signal. The binding energies (BE) for Ti 2p_3/2_ and Ti 2p_1/2_ were at 458.6 and 464.4 eV, respectively. These values are consistent with the values reported in the literature for Ti(IV) [31,32]. However, since the two signals were not symmetrical, the Ti 2p_3/2_ signal was deconvoluted. Two contributions to this signal were found, as is shown in Table 1 and in the inset in Figure 6b. The contribution at 458.7 eV is assigned to Ti(IV), as discussed previously, while the contribution at 457.2 eV is consistent with the values reported for Ti(III) [31,32]. Thus, this result is coherent with the results shown above, because the presence of Ti(III) confirms the presence of oxygen vacancies. Moreover, Figure 6c shows the O 1s signal obtained, which was also observed to be asymmetrical. The deconvolution of this signal showed the presence of three contributions, as Table 1 shows. The contribution at 529.9 eV is assigned to O^2−^ in the TiO_2_ lattice, as is reported previously [33,34]. Typically, the contributions at higher BE are not easy to assign. In our case, they can be assigned to O atoms of the medium, the diphenyl oxide, and the remains of benzylic alcohol used in the synthesis. The quantitative analysis (see Table 2) showed a higher presence of O with regard to the stoichiometric amount for TiO_2_, and also more C from the organic compounds used as the medium and as reagents in the synthesis.

### 3.2. Nanofluid Stability

**Figure 7:**
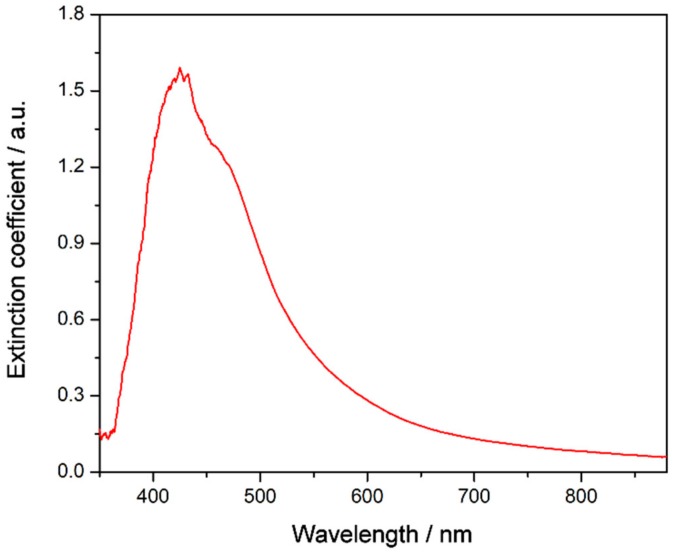
UV-vis spectrum of the TiO_2_-based nanofluid just after preparation.

**Figure 8:**
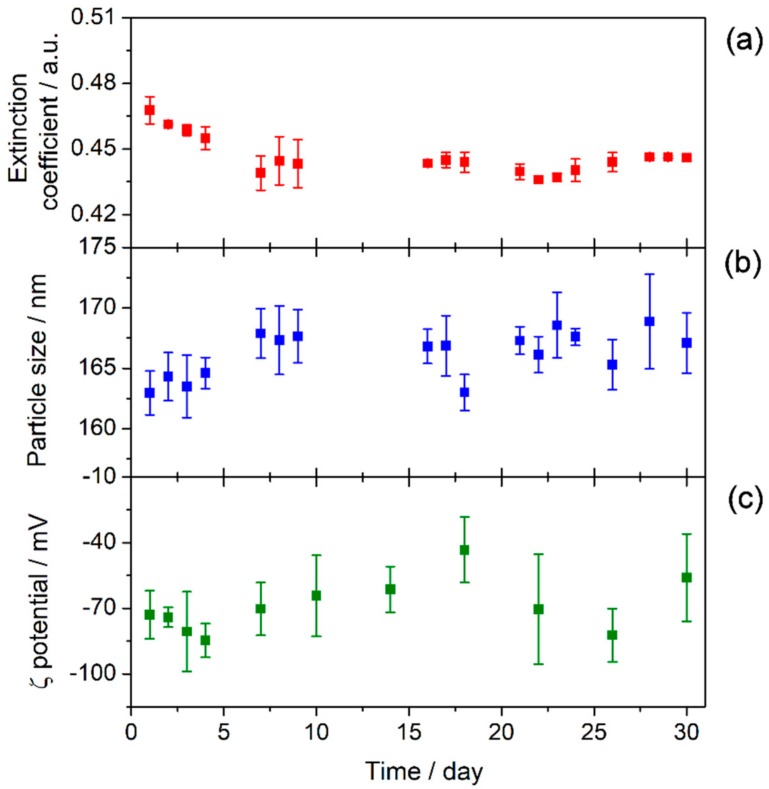
Stability of TiO_2_-nanofluid measured for 30 days: (**a**) extinction coefficient at λ = 550 nm, (**b**) particle size, and (**c**) ζ potential.

It is crucial to analyze and understand the stability of nanofluids in order to explore their practical applications, as stability plays an important role in their thermal properties. To further verify the effect of the functionalization on the stability of TiO_2_-based nanofluids, UV-vis spectroscopy, particle size measurements, and ζ potential analysis were performed. Thus, Figure 7 shows the UV-vis spectrum of the nanofluid recorded just after preparation. A wide band is visible between 350 and 600 nm that has been attributed in previous studies to photonic absorption and light scattering processes due to the suspension of nanoparticles [35,36]. Also, no chemical changes were observed in the samples with regard to the base fluid. Moreover, UV-vis spectra were recorded for one month, registering several spectra each day. Thus, Figure 8a shows the extinction coefficient at λ = 550 nm for one month. This wavelength was chosen because the effects of light scattering and photonic absorption related to the concentration of nanoparticles dispersed into the base fluid take place at this wavelength [11], hence the higher the extinction coefficient, the greater the presence of nanoparticles inside the fluid. The extinction coefficient of the nanofluid decreased slightly until the sixth day, after which it remained stable until the thirtieth day after preparation. Therefore, the results showed that the sedimentation and agglomeration processes were reduced and controlled by the preparation of the TiO_2_-nanofluid using the one-step method. 

**Figure 9:**
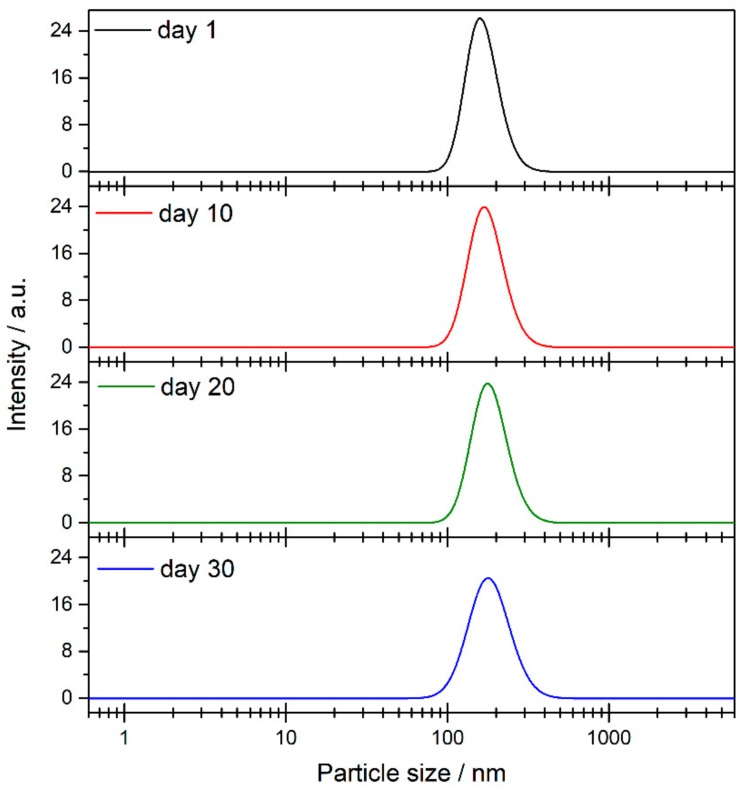
Results of particle size and size distribution from dynamic light scattering (DLS) technique.

The sedimentation process is closely linked with the size of the agglomerates in the medium. For this reason, nanoparticle size was measured by using the DLS technique. Figure 8b shows the evolution of the particle size in the nanofluid over thirty days. The particle size values vary between 160 and 170 nm for one month, meaning that the TiO_2_ nanoparticles were highly stable. Agglomeration probably does not occur due to the presence of species from the solvent surrounding the nanoparticles, as is shown from the FTIR results. Also, Figure 9 shows distribution size recorded on specific days. No changes were observed in distribution size; thus, agglomeration did not occur. The magnitude of the ζ potential gives additional information about the potential stability of the colloidal system, as this measures the charge at the slipping plane of the nanoparticle. A highly negative or positive ζ potential implies a strong repulsion between the nanoparticles. Some authors have reported that the stability threshold for colloidal solutions is a |ζ| potential value higher than ±30 mV [37]. Figure 8c shows the ζ potential values for the TiO_2_-nanofluid measured for one month. The average ζ potential fluctuated between −40 and −80 mV. These results suggest that the surface of the nanoparticles may be covered by a charged layer, which impedes the agglomeration and consequent flocculation of the nanoparticles. Thus, it can be determined that repulsion between nanoparticles prevents the formation of larger particles, as the DLS technique shows. Hence, the nanoparticles are not heavy enough to precipitate because the push force of the fluid is higher than the weight of the nanoparticle. These results are in good agreement with the high stability shown by UV-vis spectroscopy.

### 3.3. Nanofluid Performance

A nanofluid can be considered useful if it improves not only the stability of the colloidal suspension, but also its thermal properties. It is well-known that higher stability results in enhanced thermal properties. Thus, the enhancement in the efficiency of a nanofluid working under turbulent flow conditions is estimated by the ratio of the heat transfer coefficient of the nanofluid to the base fluid, given by the equation of Dittus-Boelter [38], expressed as

(2) $$\;\frac{h_{nf}}{h_{bf}}={\left(\frac{\rho_{nf}}{\rho_{bf}}\right)}^{0.8}{\left(\frac{k_{nf}}{k_{bf}}\right)}^{0.6}{\left(\frac{C_{p,nf}}{C_{p,bf}}\right)}^{0.4}{\left(\frac{\mu_{nf}}{\mu_{bf}}\right)}^{-0.4}\;$$

where *h* is the heat transfer coefficient, *ρ* is the density, *k* is the thermal conductivity, $C_{p}$ is the isobaric specific heat, and *μ* is the dynamic viscosity. The subscripts *nf* and *bf* are assigned to the nanofluid and the base fluid, respectively. Thus, the physical properties of the nanofluid and the base fluid were measured for comparative purposes. The nanofluid presented a 0.46% increase in density at room temperature (*ρ_bf_* = 1059.2 ± 0.1 kg·m^−3^; *ρ_nf_* = 1064.1 ± 0.1 kg·m^−3^). It is worth remembering that density affects the efficiency of heat transfer fluids because an increase in this property leads to a more efficient heat transfer process, so the increase generated is a positive factor in the heat transfer coefficient of nanofluids. Also, the volume fraction of the nanofluid can be estimated from density values as $\phi =\left(\rho_{nf}-\rho_{bf}\right)/\left(\rho_{np}-\rho_{bf}\right)$. Considering the density of the TiO_2_ nanoparticles as 3900 kg/m^3^ [13], the volume fraction of the nanofluid was 0.17 vol%. On the other hand, an 11.84% increase was observed in the dynamic viscosity of the nanofluid with regard to the base fluid at room temperature *(μ_bf_* = 3.80 ± 0.02 mPa·s; *μ_nf_* = 4.25 ± 0.03 mPa·s). This increase in viscosity is due to the high resistance in the nanoparticle-base fluid interface because the surface area of the smaller nanoparticles is greater [39] or due to the greater interaction between nanoparticles when they are very small [40]. Viscosity is a factor that negatively affects how nanofluids perform in a pipe, as it hinders laminar-to-turbulent regime transition and flow motion.

**Figure 10:**
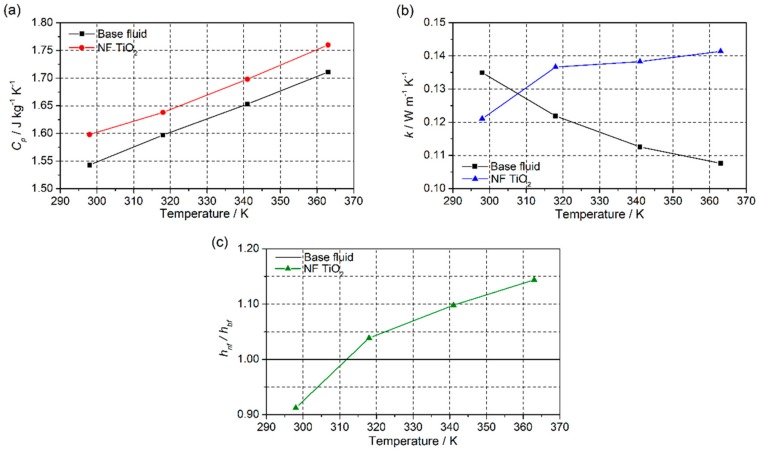
(**a**) Isobaric specific heat, (**b**) thermal conductivity, and (**c**) ratio of the heat transfer coefficient between TiO_2_-nanofluid and the base fluid.

In turn, the thermal properties of the prepared nanofluid were measured. Isobaric specific heat (*C_P_*) is one of the most important properties of nanofluids. The values of *C_P_* obtained for the TiO_2_-nanofluid and the base fluid in the temperature range between 298 and 363 K are shown in Figure 10a. A 2.8% increase in *C_P_* was observed for the nanofluid in comparison with the base fluid. A decrease in the *C_P_* of the nanofluid would be expected due to the addition of nanoparticles, as reported in previous studies [41,42,43]. The isobaric specific heat of the nanoparticles was lower than the base fluid, so, according to the prediction from the simple mixing model [44], the isobaric specific heat values for the nanofluids should be lower than those of the base fluid. However, this theory is too simple to explain the behavior of this kind of system as it does not take into account the interaction between the nanoparticles and the fluid.

The thermal diffusivity of the TiO_2_-nanofluid was measured at different temperatures using the LFA technique. The thermal conductivity of the sample was calculated using Equation (1), and the values in the range of 298 to 363 K are represented in Figure 10b. The thermal conductivity of the base fluid was also measured to check the goodness of the method used and compared with the data given by the supplier. As is observed, the nanofluid shows increased thermal conductivity at higher temperatures. The dependence of *k* on temperature for the nanofluid is totally opposite to that for the base fluid. Furthermore, the thermal conductivity of the prepared nanofluid was found to improve by 31.4% at 363 K. The increase in *k* can be attributed to several contributions, such as the stabilization of the nanoparticles, due to the possible presence of an organic layer surrounding them, which prevents the agglomeration and later sedimentation of the synthesized material. It explains the existence of a lower particle size. Hence, the fact that the number of nanoparticles suspended in the base fluid is maintained for longer leads to better heat conduction through the fluid. On the other hand, the presence of an organic layer is not only useful for the stabilization of nanoparticles, but may also be responsible of the increase of thermal conductivity. Several studies [45,46,47] suggested that the better thermal transport could be due to the presence of layers of fluid molecules surrounding nanoparticles with a lower thermal resistance. Also, it is known that the atomic structure of solids is more ordered than that of liquids, hence heat transport by lattice vibrations (i.e., phonons) is more effective; if the phonon mean free path is larger than the particle size, ballistic phonon heat conduction may be allowed. For example, as is reported by Milanese et al., the layering in Cu-based nanofluids in water plays an important role in explaining the experimental values obtained for thermal conductivity [48]. Also, in the literature, some other mechanisms are used for explaining the experimental thermal conductivity enhancement, such as Brownian motion [49], thermal boundary resistance [50], or mass difference scattering [51].

Finally, to check the heat transfer performance of the TiO_2_-nanofluid with regard to the base fluid, the results obtained were replaced in Equation (2). Thus, the ratio of the heat transfer coefficient of the nanofluid with regard to the base fluid was estimated at the temperatures at which the thermal conductivity and isobaric specific heat were measured. Figure 8c shows the values obtained. The efficiency of the heat transfer process is usually considered to have improved when $h_{nf}/h_{bf}$ is greater than 1. Thus, an enhancement in the efficiency of the heat transfer process of 14.4% at 363 K was found for the nanofluid. 

**Figure 11:**
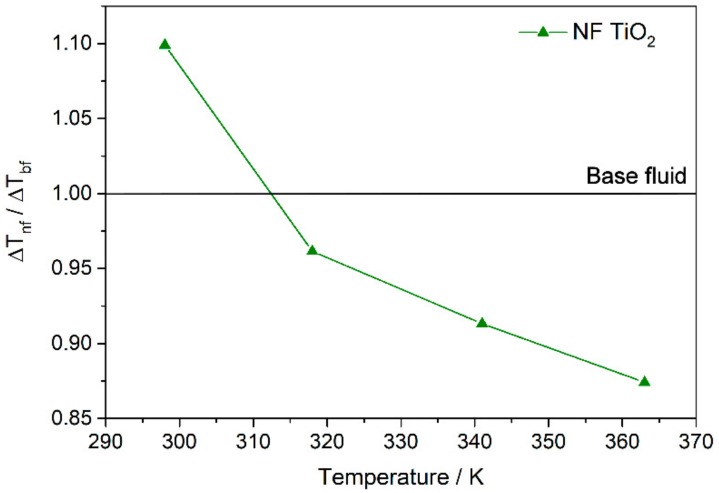
Outlet temperature in the solar collectors if nanofluids are incorporated.

Finally, the impact of the nanofluids incorporation on the efficiency of the collectors used in CSP plants can be studied from the outlet temperature. The heat flux from the surface of the receiver to the heat transfer fluid is defined as $q_{s}^{\prime \prime }=h\Delta T=h\left(T_{s}-T_{m,o}\right)$, where *T_s_* is the temperature on the surface of the pipe, and *T_m,o_* is the mean temperature of the fluid at the pipe outlet. For a constant solar irradiance of 1 sun (1000 Wm^−2^), $q_{s}^{\prime \prime }$ and *T_s_* are considered, and thus ΔT for the base fluid and for the nanofluids can be compared. Mathematically, this can be expressed as $\left(\Delta T_{nf}/\Delta T_{bf}\right)=\left(h_{bf}/h_{nf}\right)$. Then, if $\left(\Delta T_{nf}/\Delta T_{bf}\right)<1$, *T_m,o_* is higher when the nanofluid is used, and the efficiency of the collector improves, because the outlet temperature rises. So, Figure 11 shows the values of $\left(\Delta T_{nf}/\Delta T_{bf}\right)$ obtained. It is possible to observe that the use of the nanofluid prepared can lead to an increase of the outlet temperature, and therefore an increase in the efficiency of the solar collectors.

## 4. Conclusions

In this study, TiO_2_ nanoparticles were synthesized in a non-polar medium, a typical organic heat transfer fluid. The nanofluid suspension obtained from the synthesis showed a high stability and improved thermal properties.

The TiO_2_ nanoparticles synthesized were characterized using XRD, Raman spectroscopy, TEM, FTIR, and XPS. The presence of anatase phase was confirmed, and also the presence of rutile. Furthermore, the presence of a superlattice composed of anatase and rutile phases was confirmed by means of TEM. In addition, evidence of the organic species from the molecules of the medium surrounding the nanoparticles synthesized was observed. These species play an important role in providing the nanofluid formed with a high stability, which was widely characterized for one month by means of UV-vis spectroscopy and particle size and ζ potential measurements.

Also, the thermal properties of the nanofluid system formed were analyzed. Its isobaric specific heat capacity increased by 2.8%, thermal conductivity by 31.4%, and heat transfer processes by 14.4%. Thus, the nanofluid prepared could be a promising alternative to the typical HTF used thanks to its improved thermal properties and high stability, which is a result of the novel synthesis procedure followed.

## Figures and Tables

**Table 1:** Deconvolution of the Ti 2p and O 1s signals.

Ti 2p3/2			O 1s		
Peaks	B.E./eV	%	Peaks	B.E./eV	%
1	458.7	97.5	1	529.9	71.8
2	457.2	2.5	2	531.5	12.7
			3	532.5	15.5

**Table 2:** Quantitative analysis from X-ray photoelectron spectroscopy (XPS) measurements.

XPS Signal	C 1s	O 1s	Ti 2p
%	62.7	26.6	10.7

## Nomenclature

a, b, c	Lattice constants (Å)
B.E.	Binding Energy (eV)
*C_P_*	Isobaric specific heat (J kg^−1^ K^−1^)
*h*	Convective heat transfer coefficient (W m^−2^ K^−1^)
*k*	Thermal conductivity (W m^−1^ K^−1^)
$q_{s}^{\prime \prime }$	Heat flux (W m^−2^)
*T_m,o_*	Mean temperature of the fluid at the pipe outlet (K)
*T_s_*	Temperature on the surface of the pipe (K)
*α*	Thermal diffusivity (m^2^ s^−1^)
*ϕ*	Volume fraction (vol.%)
*μ*	Dynamic viscosity (Pa s)
*ρ*	Density (kg m^−3^)
**Subscripts**	
*bf*	Base fluid
*nf*	Nanofluid
*np*	Nanoparticle
